# Comparison of Two *Bacillus* Strains Isolated from the Coastal Zone in Barley (*Hordeum vulgare* L.) Under Salt Stress

**DOI:** 10.3390/plants14050723

**Published:** 2025-02-27

**Authors:** Müge Teker Yıldız, Okan Acar

**Affiliations:** Biology Department, Faculty of Science, Çanakkale Onsekiz Mart University, 17100 Çanakkale, Türkiye; oacar@comu.edu.tr

**Keywords:** barley, salt stress, plant growth-promoting rhizobacteria, antioxidant defense system

## Abstract

Salt stress is one of the most important abiotic stress factors that negatively affects sustainable crop production, agricultural productivity, and microbial life. Increasing salt stress negatively affects the growth and development of barley, posing a threat to global food security. It is now known that inoculation of plant growth-promoting rhizobacteria (PGPR) has significant potential in increasing stress tolerance and yield in agricultural products. This study focused on the effects of *Bacillus cereus* CUN6 and *Bacillus thuringiensis* SIRB2, isolated from the coastal zone and tested for their PGPR capacities, on physiological (root length, shoot length, biomass, dry weight) and biochemical (total chlorophyll, total protein, hydrogen peroxide, lipid peroxidation, peroxidase activity (POX), catalase activity (CAT)) analyses in *Hordeum vulgare* L. seedlings under salt stress. The results showed that the two bacterial inoculations alleviated the negative effects of salt stress by increasing the root-shoot length, biomass, dry weight, chlorophyll content, and total protein content in barley plants. However, *B.* *thuringiensis* increased growth and development especially in root length, biomass, and dry weight compared to *B.* *cereus.* On the other hand, *B.* *cereus* significantly increased root length, biomass, and chlorophyll content under salt stress; these increases were 17%, 5%, and 7%, respectively. *B.* *thuringiensis* chlorophyll content increased by 4% in 300 mM NaCl compared to the control. When compared in terms of the antioxidant defense system, *B.* *thuringiensis* inoculation was more effective on CAT activity, while *B.* *cereus* inoculation was more effective on POX activity. Under salt stress, *B.* *cereus* and *B.* *thuringiensis* inoculation significantly decreased H_2_O_2_ content in barley; these decreases were 16% and 10%, respectively. Additionally, TBARs content was significantly decreased by *B.* *cereus* and *B.* *thuringiensis* inoculation under salt stress; these decreases were determined as 8% and 9%, respectively, compared to the control. These results indicated that both bacterial inoculations can alleviate the salt tolerance of barley seedlings by regulating antioxidant metabolism. This research focused on the potential of *B.* *cereus* and *B.* *thuringiensis* as biofertilizers against salt stress in barley based on physiological and biochemical analysis.

## 1. Introduction

Soil salinity is the second most important abiotic stress factor in the world that restricts sustainable agriculture in arid regions by affecting agricultural productivity due to the increasing effects of global climate change [1]. When all regions of the world are examined, barley (*Hordeum vulgare* L.), one of the most important field crops grown, ranks fourth after wheat, corn, and rice in terms of production amount [2]. In addition, approximately 20% of the cultivated areas and 33% of the irrigated areas are affected by excessive salt concentrations, and this causes a 50% decrease in the agricultural production of barley, causing economic losses to producing countries [3].

High soil salinity changes the physical and chemical properties of the soil, causing ionic toxicity in plants due to the accumulation of excessive Na^+^ and CI^−^ concentrations, while preventing water and nutrient uptake and causing osmotic stress [4]. Thus, high levels of NaCl disrupt the ionic balance in plant cells, leading to the excessive production of reactive oxygen species (ROS), including superoxide anions (O_2_^−^), hydroxyl radicals (OH), hydrogen peroxide (H_2_O_2_), and singlet oxygen (O_2_) [5]. When the balance between increased ROS content in cells and detoxification is disrupted, oxidative stress occurs and the antioxidant defense system (enzymatic antioxidants, such as superoxide dismutase (SOD), catalase (CAT), ascorbate peroxidase (APX), glutathione reductase (GR), etc., or non-enzymatic antioxidants, such as ascorbic acid, glutathione, etc.), plays a crucial role in preventing oxidative damage [6]. Although plants can activate their defense mechanisms even under salt stress, they will be negatively affected by high salt stress. In the next few years, utilizing saline soils and increasing plant productivity under salt stress will be a great challenge. On the other hand, barley yield is gradually decreasing due to deficiencies in agricultural practices and unintended chemical fertilization in Türkiye. In this context, plant growth-promoting rhizobacteria (PGPR) offer an environmentally friendly and sustainable option for developing varieties resistant to salt stress, which causes serious yield losses. Thus, the use of rhizobacteria as biofertilizers is an alternative method to inorganic fertilizers or harmful chemicals [7].

Plant growth-promoting bacteria indirectly/directly trigger plant defense mechanisms. PGPR can increase the activity of antioxidant enzymes and the accumulation of osmotic regulators by inducing the antioxidant system [8]. In addition, PGPRs improve soil structure and water potential through the accumulation of exopolysaccharide (EPS) by increasing amino acids, soluble sugars, and osmolytes. Increased gibberellic acid (GA), indole-3-acetic acid (IAA), and cytokinin in the plant root zone with PGPR inoculation reduced ethylene content by ACC (1-aminocyclopropane-1-carboxylic acid) deaminase and activated signaling hormones and volatile compounds by activating induced systemic tolerance in the plant, thus defending the plant against abiotic stress limitations [9]. In previous studies, especially those conducted with the genera *Arthrobacter*, *Azotobacter*, *Bacillus*, *Caulobacter*, *Pseudomonas*, *Rhizobium*, *Serratia*, *Erwinia*, *Flavobacterium*, *Agrobacterium*, *Chromobacterium* and *Hypomycrobium*, PGPRs have been used to alleviate the negative effects of abiotic stresses [10]. Additionally recent studies have reported that PGPRs are natural biofertilizers and plant protectors and that their mechanisms need to be elucidated [11]. In salt stress conditions, the use of bacteria isolated from salty environments has become a focus [12,13]. In particular, the inoculation of PGPRs that support the resistance of agricultural products under salt stress is increasing [11]. For example, it has been reported that *Enterobacter* sp. isolated from *Psoralea corylifolia* L. promotes the growth and development of *Triticum aestivum* under salt stress [14]. In the reviewed studies, it was reported that it promotes plant growth in maize [15], tomato [16], mung bean [17], white clover [18], and wheat [19] under abiotic stress factors. On the other hand, it has been reported that salt-tolerant agricultural products use strong mechanisms, especially in the perception and signaling of salt stress, regulation of transcription, ionic homeostasis, and scavenging of reactive oxygen species. Dabrowska et al. [20] investigated the *RSH* (RelA/SpoT) gene family and salt tolerance genes in response to salinity with PGPR bacteria inoculated into *Brassica napus*. They revealed that RSH gene expression in *Brassica napus* was not affected by salt stress, but *BnRSH* gene expression was increased by inoculation of *Serratia* sp.

Despite these benefits, *B.* *cereus* has been reported as a biofertilizer in limited studies only [21]. Reports on PGPR capacities between *B.* *thuringiensis* and *B.* *cereus* in field crops under salt stress and as biocontrol agents are rare. In particular, data on the effect of bacterial inoculation on the behavior of barley under salt stress are still insufficient and fragmented. The aims of the present study were to demonstrate the efficacy of two PGPR strains in inducing salinity tolerance in barley and to highlight their capacity to enhance plant recovery under post-salinity conditions. In this context, bacterial strains were isolated from the rhizosphere of *Arthrocnemum macrostachyum* in the Çanakkale-Lapseki coastal zone of Türkiye and tested for their capacity to be PGPR. This research focused on the effects of *Bacillus thuringiensis* and *Bacillus cereus* inoculation on cultivated barley under salt stress, determining the physiological and biochemical parameters involved.

## 2. Materials and Methods

### 2.1. Sampling of Rhizospheric Soils for Bacterial Isolation

**Figure 1:**
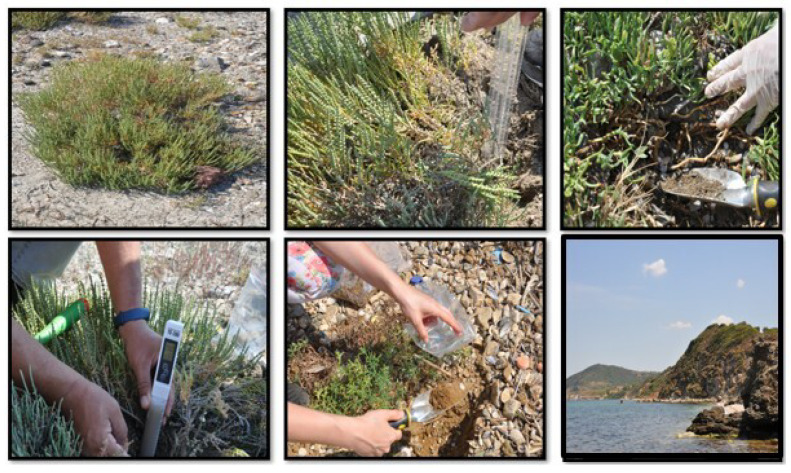
The collection of *Arthrocnemum macrostachyum* (Moric.) K.Koch. soil samples from the Lapseki coastal zone of Çanakkale province.

Plant rhizosphere soil samples were collected from the root zone of *Arthrocnemum macrostachyum* (Moric.) K.Koch. plant at a depth of 0–20 cm by random sampling from the coastal zone of the Lapseki district (40°39′29″ N, 26°92′90″ E) located in the northern part of the Çanakkale Strait opening to the Marmara Sea (Figure 1). Approximately 1 kg of soil samples was placed in paper bags and stored at 0 °C to 6 °C in the Plant Physiology Laboratory at Çanakkale Onsekiz Mart University. Then, before starting the analyses, each soil sample (1 g) was mixed thoroughly by vortexing in 9 mL of sterile water in the Microbiology Laboratory. Then, the bacterial isolates were activated with beef extract peptone (NB) solid medium. A single colony was selected in 10 mL of new NB liquid medium, centrifuged at 200 rpm at 30 °C for 12 h and the mother liquid of the isolates was obtained. It was transferred to new NB liquid medium for dilution to 1% and centrifuged at 4000 rpm and the supernatant liquid was discarded. The bacterial sediment was resuspended with sterile distilled water and serial dilutions were made up to 10^9^ CFUs (Colony Forming Units) to obtain bacterial isolate suspension for subsequent experiments. The purified isolates were stored at −20 °C in medium containing 20% glycerol.

### 2.2. Salt Resistance Screening of Isolates

The salt resistance of bacterial isolates isolated from the Çanakkale coastal zone was evaluated by monitoring their growth and development in salt-containing nutrient agar (NA) media (1% (0.172 M); 2.5% (0.43 M); 5.0% (0.86 M); 7.5% (1.29 M); 10.0% (1.72 M); and 11.62% (2 M)). In the salt resistance screening of isolates, inoculated cultures were kept in an incubator at 28 ± 2 °C for 2 days, and the isolate was considered salt-resistant when it grew in a medium containing 7.5% NaCl or more [22].

### 2.3. Morphological and Biochemical Characterization of Bacteria

The Gram staining method was used to determine the differences in cell morphology of bacterial isolates whose salt tolerance capacities were determined. For morphological characterization, dark-purple-colored microorganisms were identified as Gram (+) and light-pink-colored microorganisms were identified as Gram (−) using the Gram staining method [23].

In the oxidase test, 1% tetramethyl-p-phenylenediamine was dropped onto the colonies in agar medium during the 24 h growth phase, and colonies that gave a blue color were accepted as positive results [24].

In the catalase test, bacterial isolates were grown in nutrient media for 24 h, then incubated at 35 °C for 24 h. Then, 3% H_2_O_2_ was dropped onto the cultures and the test was considered positive for cultures with gas bubbles, and negative for cultures without gas bubbles [23].

The indole test was performed by culturing in liquid medium containing tryptophan and incubating in the oven at 37 °C for 1–5 days. Then, 0.5 mL of covac reagent was added and a bright red ring showed that the test was positive (indole formation), while a yellow ring showed that the test was negative (no indole formation) [23].

The citrate test was performed by inoculating on the surface of Simon’s Citrate Slant Agar medium and incubating in the oven at 37 °C for 2–7 days. At the end of incubation, the color of the medium, which was originally green, was evaluated as positive if turning blue, with 0.2% Bromo thymol blue used as an indicator [23].

In the Voges–Proskauer (VP) test, MR-VP (Methyl-red Voges–Proskauer) Broth medium was cultured and incubated in the oven at 37 °C for 1–7 days. After incubation, 1 mL of 40% KOH and then 3 mL of 5% alpha naphthol were added. Then, on coming into contact with air, the formation of a pink-red color indicated that the test was positive [23].

The hydrogen sulfide test involved inoculating by vertically dipping into a Triple Sugar Iron (TSI) agar tube and incubating at 37 °C for 3 days. If there was blackening along the inoculation line, it indicated the presence of H_2_S [23].

In the phenol test, a nutrient broth containing 0.5% lactose phenol indicator was inoculated into Phenol Red Lactose Broth tubes and incubated at 37 °C for 3 days. If the bacteria used lactose, the indicator color changed from red to yellow, indicating that phenol was produced [23].

### 2.4. Determination of Plant Growth-Promoting Properties

To assess PGPR properties, the plant growth-promoting hormone ACC (1-amino cyclopropane 1-carboxylic acid) deaminase enzyme activity, nitrogen fixation capacities, phosphorus solubilization capacities, indole acetic acid (IAA) production capacities, and siderophore production were evaluated.

The ACC deaminase enzyme activity of bacteria was determined by culturing on DF (Dworkin–Foster salt medium) solid medium containing ACC. Bacterial isolates were grown in 5 mL of Tryptic Soy Broth (TSB) medium with shaking at 120 rpm for 24 h at 28 °C. Then, they were centrifuged at 3000 g for 5 min, washed twice, and resuspended in 0.1 M Tris-HCl (pH 7.5). Then, cooling was performed to 40 °C. A quantity of 100 mg of ACC was dissolved in 30 mL of sterile water, filtered through a sterile filter, and added to the medium. Bacteria were taken with a sterile loop and cultured on the prepared DF Salt medium, and after 7–10 days of incubation, groups showing bacterial growth were evaluated as ACC deaminase positive [25].

To assess nitrogen fixation capacity, the medium prepared according to the literature was sterilized in an autoclave at 121 °C for 15 min. After autoclaving, it was cooled to 45 °C and left to solidify in sterile petri dishes. Bacteria taken from the stock culture were inoculated by streaking on nutrient agar medium for 2–5 days. The developing fresh bacterial colonies were incubated in a nitrogen-free medium and left to grow in an incubator set at 25–27 °C for 7–10 days. Bacteria that could grow in a nitrogen-free medium were evaluated as nitrogen-fixation-positive [26].

The phosphorus solubilization capacity was determined according to the protocol described by Mehta and Nautiyal [27]. The isolates were first incubated in NBRIP (National Botanical Research Institute’s Phosphate) liquid medium for 3 days. At the end of incubation, the isolates were inoculated with 10 µL of NBRIP solid medium containing Ca_3_(PO_4_)_2_ and bromophenol blue as a pH indicator, and spot cultivation was performed and the isolates incubated at 30 °C for 3 days. At the end of incubation, transparent zones forming around the bacteria were accepted as positive results and phosphorus solubilization coefficients were calculated by the following equation:

(1) %Phosphorus solubilization coefficient=Transparent zone diameter/Bacterial colony diameter

The amount of IAA production was tested by colorimetric analysis [28]. To determine the amount of IAA production, bacteria cultures incubated for 7 days in nutrient broth medium containing 0.1% tryptophan were centrifuged at 10,000 rpm for 10 min and 2 mL of the supernatant was taken. This supernatant was mixed with 2 drops of orthophosphoric acid and 4 mL of Salkowski reagent and the amounts of IAA were determined at 530 nm wavelength on a UV spectrophotometer (Thermo Scientific Genesys 10S VIS, Madison, WI, USA) after 30 min in the dark [29].

The siderophore production capacities were determined by spot inoculation on Chrome Azurol S agar (CAS) medium recommended by Schwyn and Neilands [30]. Then, the medium was incubated at 28 ± 2 °C for 4 days. A yellow-orange color formed around the bacteria at the end of incubation was evaluated as a positive result and the formed zone diameter (mm) was measured.

### 2.5. Molecular Identification of Bacterial Isolates

Two bacterial strains isolated from the coastal zone of Çanakkale Lapseki and evaluated for their plant growth-promoting properties were selected and incubated in Nutrient Broth (NB) medium for molecular identification. The incubated bacteria were extracted according to the modified method. Bacterial colonies were suspended in sterile 50 µL of sterile deionized H_2_O. The cell suspension was heated in a water bath at 97 °C for 10 min, then the cell lysate was strained 15,000 g 10 min and the upper layer containing the DNA was recovered. Then, a fragment of the 16S rDNA was amplified by PCR using bacterial primers 27F (5′-TCCGTAGGTGAACCTGCGG-3′) and 1492R (5′-TCCTCCGCTTATTGATATGC-3′). The PCR buffer contained, 0.5 mM MgCl_2_, 2.5 U Taq DNA polymerase, 0.25 mM dNTP (deoxynucleoside triphosphate), 0.5 µM primers, and approximately 5 ng of bacterial genomic DNA [31].

Analyses of the 16S rDNA gene sequence were performed by applying BLAST (version 2.7.1) to NCBI (National Center for Biotechnology Information), and the sequences in the databases were determined as *Bacillus thuringiensis* SIRB2 (Similarity rate 100%, Accession number: MT510408.1) and *Bacillus cereus* CUN6 (Similarity rate 100%, Accession number: MH997515.1) https://blast.ncbi.nlm.nih.gov/Blast.cgi, accessed on 12 December 2023 [32].

### 2.6. Plant Inoculation and Growth

**Figure 2:**
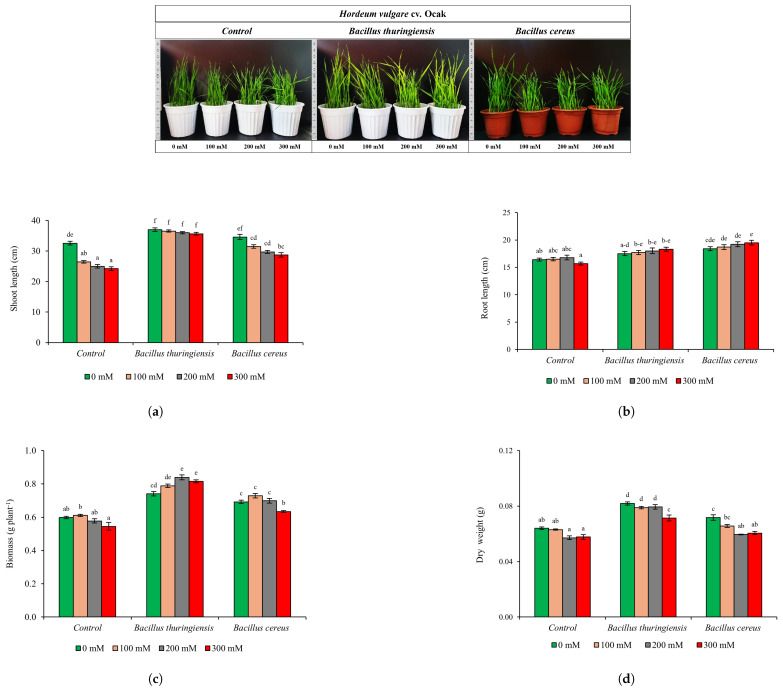
The effects of *B.* *thuringiensis* and *B.* *cereus* inoculation on cultivated barley (*H.* *vulgare* L. cv. Ocak) under salt stress (0, 100, 200, 300 mM NaCI) on shoot length (**a**), root length (**b**), biomass (**c**), dry weight (**d**). (Mean values followed by different letters are significantly different at *p* ≤ 0.05).

Seeds of *Hordeum vulgare* L. varieties were obtained from the Thrace Agricultural Research Institute (cv. Ocak). The surface sterilization of the barley seeds was performed by immersion in 70% ethanol for 2 min, in 1.2% sodium hypochlorite for 10 min, and finally, 10 times washing with distilled water. A bacterial suspension of the *Bacillus cereus* CUN6 and *Bacillus thuringiensis* SIRB2 isolate at concentrations of 10^9^ CFU mL^−1^ was inoculated into sterilized barley seeds by shaking for 30 min and dried with blotting paper for 1 h in the dark [7]. Then, plants inoculated with *B.* *cereus* and *B.* *thuringiensis* were grown in pots containing perlite with full-strength Hoagland nutrient solution at 65–70% relative humidity, at 22°C and a 16 h light/8 h dark photoperiod for 21 days in a plant growth cabinet [33]. The experiment was carried out with 3 replications randomly. The plants were then exposed to full-strength Hoagland solution, containing 0 mM, 100 mM, 200 mM, and 300 mM NaCl. On the 14th day following the salt stress, the plants were harvested, images were taken before and after the application, and the tissues were stored at −20 °C (Figure 2).

### 2.7. Plant Physiological and Biochemical Analysis

The shoot and root lengths of the barley seedlings were determined by measuring the green part of the seedling from the tip to the root with a ruler, and the shoot length (cm) was determined by measuring the root length (cm) of the seedling with a ruler.

For biomass measurement, three seedlings were taken from each group and weighed on a precision scale (g plant^−1^). To determine dry weight, three seedlings from each group were weighed on a precision scale and dried in an oven at 80 °C for 24 h (g plant^−1^).

The total chlorophyll content of barley leaves was determined using a total chlorophyll meter (Minolta, SPAD-502, Osaka, Japan) using different leaves of the seedlings with 15 replicates [34].

Total protein analysis of barley seedlings was performed by homogenization with 50 mM Na-P buffer (pH 7.8) containing 1 mM EDTA and then centrifuging. Then, the homogenates were mixed with protein reagent (Coomassie Brilliant Blue G 250, ethanol (50 mL), and ortho-phosphoric acid (100 mL)) and reading was performed at 595 nm using a spectrophotometer device. Finally, the values read were put into the formula in the standard curve graph and the total protein content (mg g^−1^) was calculated [35].

The hydrogen peroxide content (H_2_O_2_) of barley seedlings was determined spectrophotometrically at 550–800 nm (µ mL^−1^) by mixing fresh leaf tissues with 3 mL H_2_SO_4_ and cold acetone buffer and centrifuging. The last stage was determined spectrophotometrically at 550–800 nm (µg mL^−1^) by mixing the supernatant with e-FOX reading buffer (H_2_SO_4_, distilled water, ferrous ammonium sulfate, xylenol orange, sorbitol, and ethanol) [36].

The lipid peroxidation content (TBARs) of barley seedlings was determined according to the method of by Madhava Rao and Sresty [37]. First, the leaf samples were homogenized in 2.5 mL of trichloroacetic acid (TCA 0.1%) and the supernatant liquid obtained was mixed with 4 mL of trichloroacetic acid (20% TCA) containing thiobarbituric acid (TBA 0.5%) and then exposed to 95 °C in a hot water bath for 30 min. Finally, the mixture was cooled and measured at 532–600 nm in a spectrophotometer (nmol g^−1^).

The catalase activity (CAT) of barley seedlings was calculated according to the method by Bergmeyer [38]. The supernatant used in the protein analysis was mixed with reading buffer and 3% H_2_O_2_. Activity was determined at 240 nm in a spectrophotometer, and was calculated as μmol H_2_O_2_ consumed per minute.

The peroxidase activity (POX) of barley seedlings was measured by homogenization with 2 mL of cold sodium acetate buffer (C_2_H_3_NaO_2_) (pH 6.5) and reading was carried out at 300 nm with a spectrophotometer using sodium acetate buffer, pyrogallol, and H_2_O_2_ solutions [39].

### 2.8. Statistical Analysis

The data obtained in our study were analyzed using one-way ANOVA with the means ± standard error of five repetitions, and the Tukey test was used to evaluate the effects of the physiological and biochemical variables in barley using SPSS 27.0 software. In the comparisons performed, results were considered to be significantly different when *p* ≤ 0.05. In addition, for the correlation between the variables of physiological and biochemical analyses of two *Bacillus* species inoculated into barley under salt stress conditions, Principal Component Analysis (PCA) was performed using biplot and HeatMap Pearson correlation analysis with OriginPro application 2025 (Originlab, Northampton, MA, USA, https://www.originlab.com/, accessed on 9 February 2025) based on the mean value.

## 3. Results

### 3.1. Morphological and Biochemical Characterization of Bacteria

Isolates were obtained from the root zone of *Arthrocnemum macrostachyum* (Moric.) K.Koch, a halophyte plant in the coastal zone of Çanakkale Lapseki, Türkiye (Figure 1). The two bacteria strains were characterized as *B.* *cereus* CUN6 and *B.* *thuringiensis* SIRB2 by 16S rRNA sequence analysis, considering their salt stress capacities and PGPR properties. The isolated bacteria were evaluated according to their growth in 1% (0.172 M); 2.5% (0.43 M); 5.0% (0.86 M); 7.5% (1.29 M); 10.0% (1.72 M); and 11.62% (2 M) NaCl. Accordingly, *B.* *cereus* and *B.* *thuringiensis* strains were selected as tolerant to high salt since they grew by up to 11.6% in 2 M NaCl. When the cell morphology was examined, it was determined that the *B.* *cereus* CUN6 and *B.* *thuringiensis* SIRB2 bacterial isolates were positive in Gram staining. In addition, when examined in terms of cell shape, *B.* *cereus* CUN6 and *B.* *thuringiensis* SIRB2 isolates were determined to be bacilli (Table 1).

When the biochemical characterizations were examined, it was determined that *B.* *cereus* oxidized 1% tetramethyl-p-phenylenediamine reagent in the presence of oxidase enzyme, creating a purple-blue color and giving positive results in the oxidase test, while *B.* *thuringiensis* isolate gave negative results (Supplementary Materials, Figure S1) (Table 1). On the other hand, the catalase test of the *B.* *cereus* isolate was negative, and *B.* *thuringiensis* was positive when reacting with H_2_O_2_, forming gas bubbles. In the indole test, both isolates were determined to be positive by breaking down the tryptophan amino acid with the addition of Kovacs reagent, forming a red ring (Supplementary Materials, Figure S1). In the citrate test, *B.* *cereus*, which used citrate as a carbon source and formed a blue color, was evaluated as positive, while *B.* *thuringiensis*, which turned green, was determined to be negative. In the phenol test, only *B.* *cereus*, which turned from red to yellow and produced gas, was determined to be positive. In the VP test, *B.* *cereus*, which gave a pink-red color with the dropping of KOH, was evaluated as positive, while *B.* *thuringiensis* was determined to be negative. In the H_2_S test, ferric ammonium reacted with citrate and the bottom color of the tube was evaluated as red (bacteria fermenting glucose) or the bottom color was evaluated as yellow (bacteria fermenting glucose and breaking down lactose) (Table 1) (Supplementary Materials, Figure S1).

### 3.2. Identifying Plant Growth-Promoting Properties of Bacteria

In our study, it was determined that both bacterial isolates had low phosphorus solubilization capacities (Table 2). Siderophore production capacities were evaluated by calculating the zone diameter of the color formed by siderophores on iron-containing CAS agar (Table 2) (Supplementary Materials, Figure S2). In this context, *B.* *cereus* siderophore production was found to be higher than *B.* *thuringiensis* with a zone diameter of 24 mm. The ACC deaminase enzyme activity of both bacterial isolates was found to be positive (Table 2) (Supplementary Materials, Figure S2). It was determined that both isolates fixed nitrogen from the isolates incubated in nitrogen-free liquid medium to determine nitrogen-fixing isolates (Table 2) (Supplementary Materials, Figure S2). The amount of IAA production of bacteria was determined by evaluating as positive the isolates with pink-red color change with Salkowski reagent in nutrient broth medium containing tryptophan (Supplementary Materials, Figure S2). In addition, the amount of IAA of the isolates at 530 nm was determined as 75.0 µg mL^−1^ for *B.* *cereus* (Table 2) (Supplementary Materials, Figure S2).

### 3.3. Physiological and Biochemical Analysis in Barley Under Salt Stress

#### 3.3.1. Physiological Analysis

In this study, physiological and biochemical changes caused by two *Bacillus* isolates selected according to their salt tolerance capacities and PGPR criteria in barley (*H.* *vulgare* L. cv. Ocak) under salt stress were determined. In the Control group (non-inoculation), the negative effect of increasing salt concentrations suppressed growth parameters in barley (Figure 2). In this group, shoot length decreased by 19%, 23%, and 26% at 100 mM, 200 mM, and 300 mM NaCl, respectively (*p* ≤ 0.05) (Figure 2a). *B.* *thuringiensis* inoculation reduced shoot length by 4% only at 300 mM NaCl compared to 0 mM NaCl (*p* ≤ 0.05). On the other hand, *B.* *cereus* inoculation decreased shoot length by 17%, especially at 300 mM NaCl compared to 0 mM NaCl (*p* ≤ 0.05). It was determined that root length improved against increasing salt stress with both bacterial inoculations compared to the Control group (Figure 2b). Especially, root lengths in barley under 300 mM NaCl stress increased by 5% and 7% with *B.* *thuringiensis* and *B.* *cereus* inoculations, respectively (*p* ≤ 0.05) (Figure 2b). Both bacterial inoculations caused a significant increase in biomass compared to 0 mM NaCl application in the Control group (*p* ≤ 0.05) (Figure 2c). Furthermore, biomass did not change with increasing salt stress with *B.* *cereus* inoculation, while *B.* *thuringiensis* inoculation increased biomass by 6%, 13%, and 10%, respectively (*p* ≤ 0.05) (Figure 2c). Dry weight decreased in the Control group with all NaCl applications in Ocak under salt stress (Figure 2d). *B.* *thuringiensis* inoculation increased dry weight by 25%, 39%, and 24%, respectively, in all NaCl applications compared to Control plants (*p* ≤ 0.05) (Figure 2d). On the other hand, *B.* *cereus* inoculation increased by 4–5% in all NaCl applications (*p* ≤ 0.05) (Figure 2d) (Supplementary Materials, Tables S1 and S2).

#### 3.3.2. Biochemical Analysis

Total protein content was significantly increased at all concentrations in the Control group (*p* ≤ 0.05) (Figure 2a). On the other hand, it was found that *B.* *thuringiensis* and *B.* *cereus* inoculations in barley under 300 mM NaCl stress reduced the protein content by 25% and 23%, respectively, compared to 0 mM NaCl (*p* ≤ 0.05) (Figure 2a). Moreover, the amount of protein was significantly reduced by two-fold with *B.* *cereus* application compared to the Control group (*p* ≤ 0.05) (Supplementary Materials, Tables S3, S4 and S7). The amount of chlorophyll is an indicator of the healthy functioning of photosynthetic activity.

Total chlorophyll content decreased significantly in the Control group at all salt concentrations (*p* ≤ 0.05) (Figure 2b). Surprisingly, both bacterial inoculations increased the amount of chlorophyll with increasing salt concentrations (*p* ≤ 0.05). *B.* *thuringiensis* and *B.* *cereus* inoculations increased total chlorophyll amount by 66% and 69%, respectively, compared to the 300 mM NaCl application in the Control group (*p* ≤ 0.05) (Figure 2b) (Supplementary Materials, Tables S3, S4 and S7).

**Figure 3:**
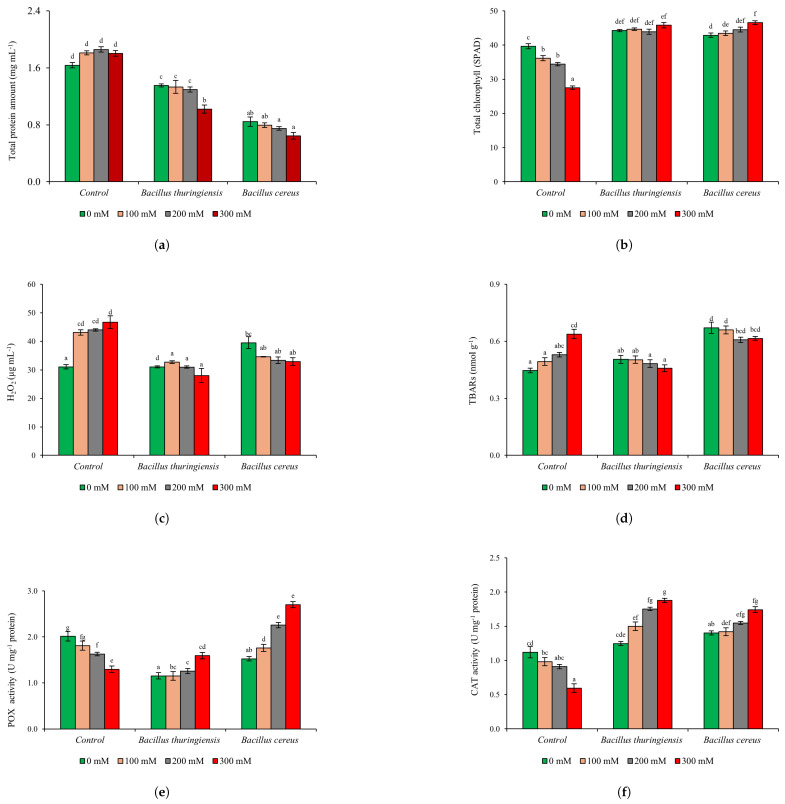
The effects of *B.* *thuringiensis* and *B.* *cereus* inoculation on cultivated barley (*Hordeum vulgare* L. cv. Ocak) under salt stress (0, 100, 200, 300 mM NaCI) on total protein content (**a**), total chlorophyll content (**b**), hydrogen peroxide content (H_2_O_2_) (**c**), lipid peroxidation content (TBARs) (**d**), peroxidase activity (POX) (**e**), catalase activity (CAT) (**f**) (Control: non inoculation). (Mean values followed by different letters are significantly different at *p* ≤ 0.05).

In our study, H_2_O_2_ content increased by 39%, 41%, and 50% with increasing salt stress in the non-inoculation group compared to 0 mM NaCl (*p* ≤ 0.05) (Figure 3c). On the other hand, H_2_O_2_ content was found to be decreased with *B.* *thuringiensis* and *B.* *cereus* inoculation compared to 0 mM NaCl application. Accordingly, both bacterial inoculations decreased by 10% and 16% at 300 mM NaCl, respectively (*p* ≤ 0.05) (Figure 3c) (Supplementary Materials, Tables S3, S4 and S7).

TBARs levels dropped in all salt stress treatments with both bacterial inoculations, whereas in the Control group they rose as salt stress increased (*p* ≤ 0.05) (Figure 3d). Accordingly, it was determined that *B.* *thuringiensis* inoculation decreased lipid peroxidation by 9% and 28% in 200 mM and 300 mM NaCl applications, respectively, compared to the concentration in the Control group (*p* ≤ 0.05). In contrast, *B.* *cereus* inoculation reduced lipid peroxidation by 4% only at 300 mM NaCl (*p* ≤ 0.05) (Figure 3d) (Supplementary Materials, Tables S3, S4 and S7).

In the Control group, with increasing salt stress, POX activities gradually decreased by 10%, 19%, and 36% at 100 mM, 200 mM, and 300 mM NaCl, respectively (*p* ≤ 0.05) (Figure 3e). Furthermore, *B.* *thuringiensis* inoculation was found to increase POX activity by 23% compared to Control, and by 38% compared to 0 mM NaCl (*p* ≤ 0.05). Especially at 300 mM NaCl. *B.* *cereus* inoculation gradually increased POX activities at all salt concentrations (*p* ≤ 0.05). Moreover, POX levels at 300 mM NaCl were dramatically increased by two-fold compared to Control with *B.* *cereus* inoculation, and by 77% compared to 0 mM NaCl (*p* ≤ 0.05) (Figure 3e) (Supplementary Materials, Tables S3, S4 and S7).

In the Control group, CAT activities decreased gradually with increasing salt stress, similar to POX levels, by 12%, 19%, and 47% at 100 mM, 200 mM, and 300 mM NaCl, respectively (*p* ≤ 0.05) (Figure 3e). CAT levels increased gradually with *B.* *thuringiensis* inoculation. However, both bacterial inoculations increased CAT levels dramatically by three-fold, especially at 300 mM NaCl (*p* ≤ 0.05) (Figure 3f) (Supplementary Materials, Tables S3, S4 and S7).

### 3.4. Correlations Between Physiological and Biochemical Characteristics

**Figure 4:**
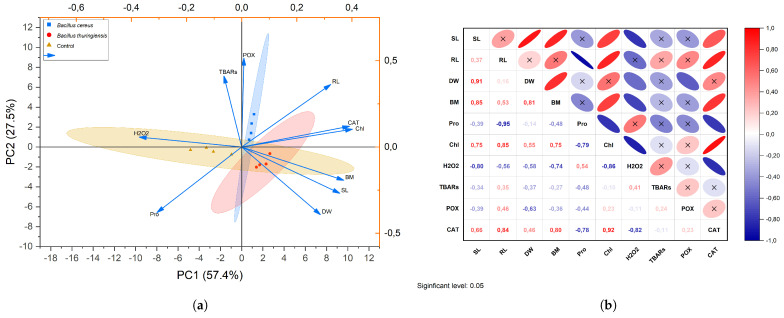
PCA biplot analysis (**a**) and HeatMap of correlations (**b**) of physiological and biochemical variables examined by inoculation of *B.* *thuringiensis* and *B.* *cereus* onto barley under salt stress (SL: Shoot lenght; RL: Root lenght; BM: Biomass; DW: Dry weight; Pro: Total protein content; Chl: Total chlorophyll content; H_2_O_2_: Hydrogen peroxide content; TBARs: Lipid peroxidation content; POX: Peroxidase activity; CAT: Catalase activity).

Principal Component Analysis (PCA) was performed to distinguish between physiological and biochemical analyses of *B.* *thuringiensis* and *B.* *cereus* inoculation on barley grown under salt stress. In the first biplot analysis, PC1 and PC2 explained the variability of the result by 57.41% and 27.50%, respectively (*p* ≤ 0.05) (Figure 4a). A PCA biplot analysis performed for physiological and biochemical traits showed a good contribution in terms of bacterial performance when depicting the relationships between the analyses under stressful conditions of the studied barley plant. For the physiological parameters, PCA biplots showed a strong positive correlation between SL, RL, BM, Chl, and DW, while PCA biplots of the enzymatic biochemical traits CAT and POX showed a strong positive relationship (*p* ≤ 0.05) (Figure 4a). On the other hand, SL, DW, RL, BM, Chl, and CAT, POX activities were found to be closer, while H_2_O_2_ and TBARs were found to be negatively clustered (*p* ≤ 0.05) (Figure 4a) (Supplementary Materials, Figure S3, Tables S5 and S6). These strongly positive/negative correlated traits can be considered to contribute maximally to yield output when compared with each other.

Pearson correlation was performed for HeatMap analysis of the physiological and biochemical traits of *B.* *thuringiensis* and *B.* *cereus* when inoculated on barley plant under salt stress (Figure 4b). The correlation between the physiological and biochemical parameters was significantly and positively correlated, as highlighted in the red color circle in Figure 4b. In particular, H_2_O_2_ content and TBARs content showed a significant positive correlation (red circle) with oxidative stress biomarkers together (*p* ≤ 0.05) (Figure 4b). In addition, SL, RL, BM, Chl, and DW showed a significant positive correlation (red circle) with physiological parameters and POX and CAT activity (*p* ≤ 0.05). On the other hand, H_2_O_2_ and TBARs content showed a negative correlation (blue circle) with SL, RL, BM, Chl, DW, CAT, and POX (*p* ≤ 0.05) (Figure 4b). However, the activities of antioxidant enzymes (POX and CAT) were also significantly positively correlated with plant growth, as shown in light red color (Figure 4b) (Supplementary Materials, Figure S3, Tables S5 and S6).

## 4. Discussion

This study aimed to compare the physiological and biochemical effects of two *Bacillus* isolates selected according to their PGPR properties in alleviating the negative effects of salt stress on barley cultivars.

Climate change caused by global warming negatively affects the seasons, increasing temperatures and decreasing precipitation rates, causing salt accumulation on the soil surface, thus deteriorating the productivity of agricultural lands. As the salt concentration in the soil increases, early seed germination and seedling and root development are negatively affected, thus limiting agricultural productivity [40]. The accumulation of ions that cause salt stress in the soil and plant tissues over time causes inhibitory effects on plant growth [41]. Salt stress disrupts the balance of cellular ions in plants in the future, negatively affecting various biological processes, including photosynthesis and cell division. This disruption generally leads to a decrease in chlorophyll levels, limiting photosynthetic efficiency and restricting plant growth [42].

In this study, it was shown that in the group without bacteria application, increasing salt stress negatively affected the growth parameters of barley. On the other hand, while the chemicals applied to the soil for sustainable agriculture today contribute to agricultural productivity by killing pathogens and weeds, irreversible damage is caused to the environment and plant ecosystem. In addition to this damage, the effects of pesticides on the ecosystem have started to negatively affect humans. Therefore, attention to plant–bacteria relationships has high potential for application in the future for both plant and human health to ensure sustainable life. The use of PGPRs as bioprimers (seed coating) in soils salinized as a result of climatic changes offers an innovative approach to sustainable agricultural practices with respect to ecosystem management. The innovative approach of use of PGPRs can reduce ethylene levels by triggering ACC production in plant tissues and promoting plant growth by increasing IAA production. [43]. In this study, both bacterial isolates increased shoot length compared to the group not inoculated with bacteria. It was determined that *B.* *thuringiensis* isolate preserved shoot length better compared to *B.* *cereus*, especially in alleviating the negative effects of salt stress. On the other hand, it was determined that root length increased in the Control group with both bacterial inoculations. One of the most important signaling pathways that promote plant growth and development and plays a role in combatting environmental stresses involves phytohormones [44]. PGPRs, which positively affect plant development, are known to produce various hormones as signaling molecules in the root region. In particular, the PGPR family, which includes *Bacillus*, *Pseudomonas*, and *Bradyrhizobium* species, has been found to produce plant growth hormones, such as IAA, GA, and ABA [45]. It has been reported that *Azorhizobium caulinodans*, *B.* *japonicum*, *Rhizobium japonicum*, *R.* *leguminosarum*, *R.* *meliloti*, *R.* *phaseoli*, *R.* *trifolii* and *Sinorhizobium meliloti* bacteria produce IAA via indole-3-pyruvic acid and contribute to the growth and development of plants through signaling [45,46,47,48,49]. In this context, we found that the high IAA value in *B.* *cereus* (75.0 µg mL^−1^) and *B.* *thuringiensis* (65.7 µg mL^−1^) triggered the signaling pathways. In similar studies, *B.* *cereus* L10 (54.6 µg mL^−1^), *B.* *cereus* So3II (35.8 µg/mL), and *B.* *subtilis* Mt3b (36.6 µg/mL) were reported to increase root length by producing IAA, an auxin precursor, and are considered plant growth-promoting bacteria [50,51]. The current study confirms a significant positive relationship between root length and IAA production. A study by [52] found that *B.* *thuringiensis* was more effective with respect to stem length and *B.* *cereus* was more effective with respect to root length, consistent with the study. These results showed that *B.* *thuringiensis* was more effective with regard to root length and *B.* *cereus* was more effective with regard to root length.

On the other hand, increasing soil salinity continues to reduce yield by negatively affecting plant growth and development. To reduce this negative effect, salt stress-resistant PGPR applications can contribute to plant growth by alleviating salinity stress. In particular, such bacterial inoculations alleviate the negative effects of salt stress on root growth and promote root development. Thus, they can increase water absorption from the soil [53]. In this study, both *Bacillus* isolates used produced IAA at different levels, similar to the results of previous studies. As a result of the increased IAA in plants, ethylene content in the root is reduced by ACC deaminase. This activates induced systemic tolerance in the plant and protects the plant against abiotic stress through a series of mechanisms, such as activation of signaling hormones and volatile compounds [54]. On the other hand, it has been reported that the negative effects of ethylene hormone, such as inhibition of root elongation, IAA transport, and leaf senescence in plants, are eliminated by PGPRs by producing ACC deaminase enzyme [55]. Thus, studies have reported that *R.* *leguminosarum*, *R japonicum*, *R.* *gallicum*, *B.* *japonicum*, *Bradyrhizobium elkanii*, *S.* *meliloti*, and *Variovorax* sp. are ACC deaminase producers [56,57,58,59]. In this study, ACC deaminase production was found to be positive in *B.* *cereus* and *B.* *thuringiensis* (Table 2) (Supplementary Materials, Figure S2). Moreover, we can say that it increases root and shoot elongation by producing ACC deaminase and protects barley from salt stress by reducing ethylene levels. In addition, we support previous studies that suggest it increases iron uptake from the soil by increasing siderephore production in *B.* *cereus* (24 mm) and *B.* *thuringiensis* (19 mm) [60]. Thus, chlorophyll synthesis, which is central to photosynthesis, supports growth by increasing iron chelation in the photosynthetic electron chain and respiration [61].

*B. cereus* and *B.* *thuringiensis* strains have been investigated in other agricultural crops, separately, to determine direct and indirect mechanisms to enhance growth under stressful conditions in different plants. *B.* *cereus* has been found to promote plant growth in plants such as soybean (*Glycine max* L. Merr.), wheat (*Triticum aestivum* L.), cabbage (*Chinese cabbage*), the rap group (*Brassica rapa* L.), maize (*Zea mays*), potato (*Solanum tuberosum* L.), pea (*Pisum sativum* L.), and rice (*Oryza sativa* L. FARO 44) [62,63,64,65,66]. On the other hand, studies with *B.* *thuringiensis* have reported that it promotes growth in corn, cabbage, pepper, lettuce, and tomato products [67,68,69,70]. In addition, studies have reported that *Bacillus* strains induce systemic responses (ISR) in plants and control plant diseases [71,72,73,74]. In their study, El-Wakil and Essa [75] found a decrease in bacterial blight as a result of inoculation of *B.* *subtilis* and *B.* *thuringiensis* in barley against *X.* *campestris.* Moreover, Yang et al. [76] found that inoculation of *B.* *cereus* BS107 conferred systemic resistance in pepper plants.

Another result of growth suppression in barley, a glycophyte, is decrease in biomass [77]. In the Control group, an increase in biomass was detected with both bacterial inoculations compared to 0 mM NaCl application. However, while biomass did not show significant change against increased salt stress with *B.* *cereus* inoculation, *B.* *thuringiensis* inoculation alleviated the negative effect. Similarly, an increase in biomass was reported with PGPR inoculation in maize plants [43]. A positive correlation between salt tolerance and dry weight has been reported in barley [78]. Our data indicate that *B.* *thuringiensis* is more effective than *B.* *cereus* in terms of biomass and dry weight. On the other hand, the amount of protein decreased significantly with *B.* *cereus* application compared to the Control group. Total protein content was reported to decrease similarly with *Aspergillus niger* inoculation in *H.* *vulgare* [79]. In our study, total chlorophyll content decreased in barley that was not inoculated with bacteria. Surprisingly, both bacterial inoculations increased the chlorophyll amount in parallel with increasing salt concentrations. Similarly, chlorophyll content increased in PGPR-inoculated barley [80] and in beans inoculated with *B.* *thuringiensis* [81].

In addition, *B.* *thuringiensis* and *B.* *cereus* strains, which we found to have higher IAA production, have been reported to increase root and shoot growth and the chlorophyll content of barley [82]. The total chlorophyll amount is also one of the indicators of salt stress tolerance. In this way, total chlorophyll amount was significantly increased in *B.* *thuringiensis* and *B.* *cereus* inoculated plants compared to non-inoculated barley plants. These data indicate that regulating the ion content to promote chlorophyll production can increase photosynthesis and provide tolerance to salt stress [83].

Due to the disruption of the electron transport chain during photoinhibition and/or the drop in water potential, ROS such as O_2_^−^, OH^−^, and H_2_O_2_ are produced in greater quantities by plant cells under salt stress [3]. The accumulation of ROS causes oxidative stress and reacts with proteins and lipids in the plant cell, disrupting their membrane structure [84]. The amount of TBARs, a malondialdehyde (MDA) product, directly reflects the damage status of plants under salt stress [85]. The lower the TBARs content, the lower the degree of damage in plants. It has been shown that PGPR inoculation can reduce the TBARs content. MDA content decreased by 57% in inoculated barley under drought stress. However, MDA content decreased dramatically in barley inoculated with *B.* *subtilis* HAS31 [80,86]. ACC deaminase produced by PGPRs helps in alleviating the negative effects of salt stress by reducing H_2_O_2_ accumulation [87]. In this context, it was determined that H_2_O_2_ content increased with increasing salt in the group without bacteria application and, conversely, *B.* *cereus* inoculation with *B.* *thuringiensis* decreased H_2_O_2_ content. In addition, Slimani et al. [88] showed that H_2_O_2_ content decreased by 46% compared to non-inoculated barley plants. In our study, *B.* *cereus* and *B.* *thuringiensis* inoculation in barley seedlings increased the salt tolerance of an Ocak variety by reducing TBARs and H_2_O_2_ content, thus indicating that it provides protection against oxidative damage caused by salt stress. Similarly, PGPR inoculation into plants such as rice [89], chickpea [8], sunflower [90], lettuce [91], and maize [92] has been reported to increase ROS scavenging ability under abiotic stress.

In response to oxidative damage induced by ROS, plants increase the activities of antioxidant enzymes such as POX and CAT. CAT is one of the first lines of defense against ROS-induced damage in peroxisomes because it enhances the removal of ROS by catalyzing the denaturation of O_2_^−^ to O_2_ and H_2_O_2_ [31]. The POX activity catalyzes H_2_O_2_ reactions in the cell wall and apoplast to convert H_2_O and acts as a signal against abiotic stress limitations [93]. *B.* *cereus* and *B.* *thuringiensis* strains have been found to have phytohormone and ACC deaminase activity, antioxidant defense (SOD, POD, APX, CAT, GR), osmolyte accumulation, volatile compounds (VOCs), and exopolysaccharide production (EPS) in different plants [94,95,96,97]. However, there is a lack of physiological and biochemical comparisons of *B.* *cereus* and *B.* *thuringiensis* strains in barley plant under salt stress. In this context, we showed in the study that application of *B.* *cereus* and *B.* *thuringiensis* strains promoted the growth of barley plant under salt stress, as reported in other *Bacillus* species [11,74,92,94,98]. In this context, with *B.* *thuringiensis* and *B.* *cereus* inoculation, POX activity in barley was significantly higher compared to the non-inoculated group under increasing salt stress [99,100]. However, inoculation with *B.* *cereus* increased it dramatically, especially at 300 mM NaCl. Similarly, Wang et al. [101] showed that inoculation with *B.* *cereus* increased POX activity and eliminated the harmful effects of ROS species in plants. On the other hand, another H_2_O_2_ scavenger, CAT, especially with *B.* *thuringiensis* inoculation at 300 mM NaCl, exhibited the highest CAT levels compared to the other two treatment groups. The results showed that *B.* *thuringiensis* inoculation was more effective on CAT activity and *B.* *cereus* inoculation was more effective on POX activity.

PGPR enhances plant growth under stress through multiple mechanisms, including the production of osmoprotectants, such as proline, glycine betaine, and trehalose, which regulate the osmotic balance in plant cells. Many antioxidants are produced by PGPR, which limit the degree of damage caused by ROS. In addition, the induction of systemic resistance by PGPR helps the plant to withstand upcoming stress [102]. Similar research has also reported enzymatic activity results for wheat and quinoa [103,104]. Salt stress decreased the activity of antioxidant systems and increased ROS in barley. Bacterial inoculation was found to have many effective activities by absorbing reactive oxygen species and increasing the activity of antioxidants POX and CAT [105]. The results showed that bacterial inoculation triggered the antioxidant level in barley under salt stress. For example, CAT levels are high to neutralize superoxide radicals, while POX activity is elevated to reduce H_2_O_2_. This relationship was interpreted using PCA-based biplot analysis of barley performance under stressful conditions (Figure 4) [106]. PCA biplot analysis performed for both physiological and biochemical traits identified relationships among traits under stressful conditions. In this context, PCA biplots of biochemical traits, Chl, CAT, and POX, showed strong positive correlations among themselves, while PCA biplots of physiological traits SL, RL, BM, and DW, showed robust positive relationships with the investigated parameters (Figure 4). In particular, *B.* *cereus* PCA biplot analysis showed strong positive correlations with RL, Chl, and CAT while negative correlations were detected with H_2_O_2_ and TBARs (Figure 4). These positively correlated traits indicate that *B.* *cereus* inoculation contributes maximally to the yield output in barley.

## 5. Conclusions

The unthinking and excessive use of agricultural chemicals causes toxic effects to humans indirectly through plants. Therefore, environmentally friendly practices are extremely important to ensure sustainable agriculture. In this context, remarkable effects of barley-PGPR inoculation against drought stress were reported by Ferioun et al. [80], Talaat et al. [107], and Slimani et al. [88]. This study showed that the plant growth-promoting bacteria, *B.* *thuringiensis* and *B.* *cereus*, which were isolated from the Çanakkale Lapseki coastal zone and were determined to have multifunctional PGPR properties, played a very important role in the physiological (root-shoot length, biomass, dry weight) and biochemical (total chlorophyll, total protein, hydrogen peroxide, lipid peroxidation, POX, CAT) parameters of barley under salinity stress. Accordingly, when comparing *B.* *thuringiensis* and *B.* *cereus*, which promote growth by increasing salt tolerance, it was determined that they both significantly improved the growth parameters of barley and provided protection from ROS damage by increasing salt stress-induced POX and CAT activities and reducing the amount of TBARs and H_2_O_2_. The results show that *B.* *cereus* is as effective as *B.* *thuringiensis*, with great potential for promoting barley growth and improving salt tolerance. On the other hand, it has been reported that *Bacillus* species can be used as biocontrol agents or biofertilizers by promoting plant growth based on their exopolysaccharide, siderophore, phytohormone, and phosphorus-solubilizing properties [58,108,109]. Therefore, *B.* *cereus* CUN6 may have the potential to be used as a biocontrol agent and/or biofertilizer against salt stress. On the other hand, there are some concerns about the inoculation of PGPRs into agricultural products, especially in field applications. The only way to minimize these concerns is to continue interdisciplinary research to understand microbial communities and plant–bacteria interactions. Moreover, the use and commercialization of PGPRs resistant to environmental stresses will now be facilitated through the contribution of many studies. Further, agricultural lands with high soil salinity should be better examined in terms of productivity. The mechanisms, in terms of the signaling pathways at biochemical and molecular levels, by which bacteria living in the saline soil rhizosphere perform as PGPR should be investigated in depth, and suitable bacterial candidates should be selected to continue to contribute to sustainable agriculture.

## Figures and Tables

**Table 1:** Morphological and biochemical characterization of *B.* *cereus* and *B.* *thuringiensis*.

	*Bacillus cereus* CUN6	*Bacillus thuringiensis* SIRB2
Pigment	Orange	Orange
Gram Stain	+	+
Morphology	Bacil	Bacil
Oxidase Test	+	−
Catalase Test	−	+
Indole Test	+	+
Citrate Test	+	−
Phenol Test	+	−
VP Test	+	−
H_2_S Test	Y ^SC^	R ^SC^
	Y ^DC^	R ^DC^

^SC^ Surface color, ^DC^ Deep color, +: Positive, −: Negative, R: Red, Y: Yellow.

**Table 2:** Plant growth-promoting properties of *B.* *cereus* and *B.* *thuringiensis*.

Isolate	NBRIP (mm)			CAS (mm)	ACC	Nitrogen Fixation	IAA
	Zone Diameter	Colony Diameter	Phosphorus Dissolution	Zone Diameter	Reproductive	Reproductive	530 nm
*Bacillus cereus* CUN6	12	7	1.71	24	+	+	75.0
*Bacillus thuringiensis* SIRB2	18	16	1.12	19	+	+	65.7

## Data Availability

The original contributions presented in the study are included in the article; further inquiries can be directed to the corresponding author.

## Supplementary Materials

The following supporting information can be downloaded at: https://www.mdpi.com/article/10.3390/plants14050723/s1, Figure S1: Morphological and biochemical characterization of *B.* *cereus* and *B.* *thuringiensis*; Figure S2: Plant growth-promoting properties of *B.* *cereus* and *B.* *thuringiensis*; Figure S3: The PCA biplot analysis of physiological and biochemical data in *B.* *cereus* and *B.* *thuringiensis* inoculated barley plants; Table S1: Physiological parameter variance homogeneity tests in PGPR-inoculated barley plants; Table S2: The ANOVA of measured physiological parameters (F values); Table S3: Biochemical parameter variance homogeneity tests in PGPR-inoculated barley plants; Table S4: The ANOVA of measured biochemical parameters (*F values*); Table S5: The PCA percentage of variance (%) and cumulative (%); Table S6: The correlation table of physiological and biochemical analysis data; Table S7: *T*-test and effect values of physiological and biochemical analysis data.
