# Supplementary material for: Comparison of Two Bacillus Strains Isolated from the Coastal Zone in Barley (Hordeum vulgare L.) Under Salt Stress
Source: Plants (Basel). 2025 Feb 27;14(5):723. doi: 10.3390/plants14050723 (PMC11902031; doi:10.3390/plants14050723)
Supplement: Supplementary file 1 [file plants-14-00723-s001.zip › plants-3471251-supplementary.pdf]

Supplementary Material

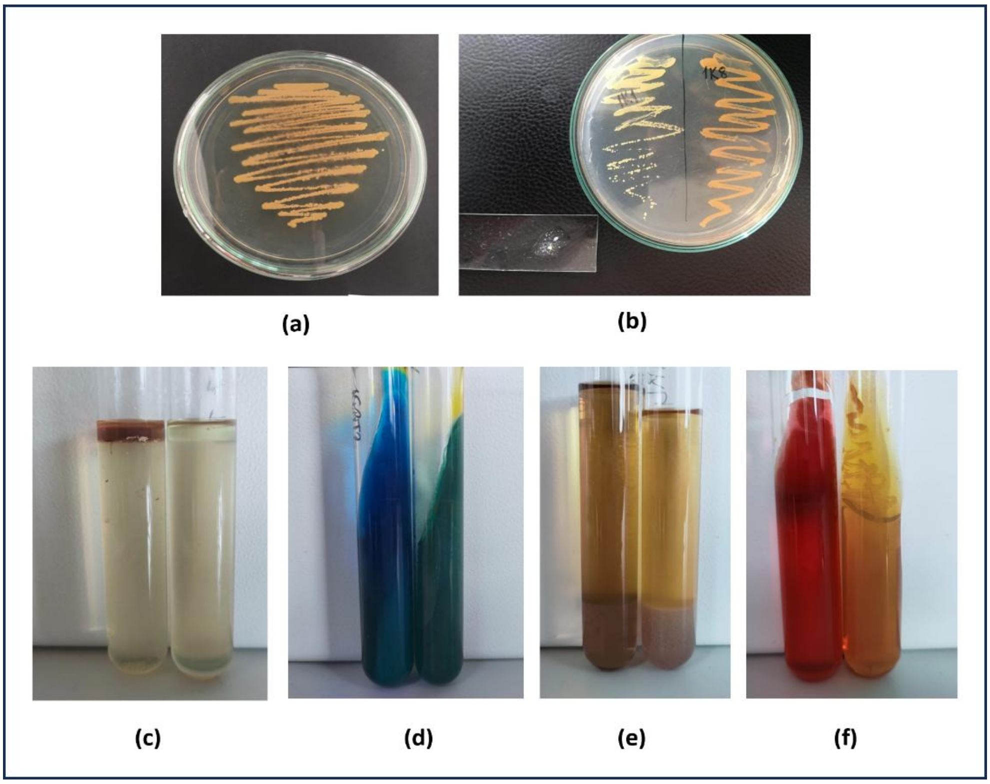

Figure S1. Morphological and biochemical characterization of *B. cereus* and *B. thuringiensis* (**a**:Pigment; **b**: Catalase Test; **c**: Indole Test; **d**: Citrat Test; **e**: VP Test; **f**: H_2S Test).

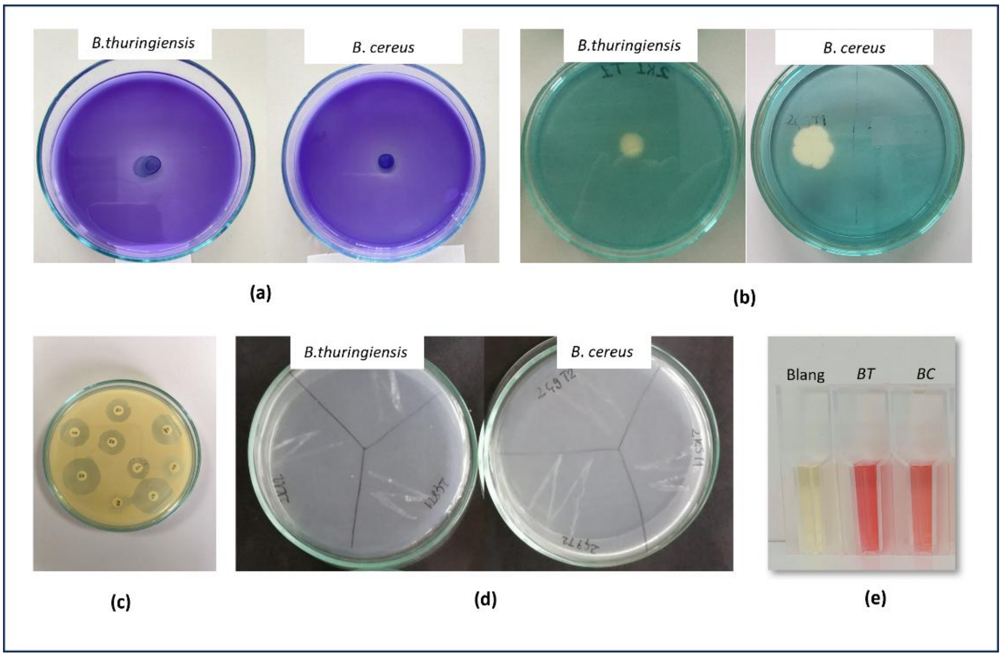

Figure S2. Plant growth promoting properties of *B. cereus* and *B. thuringiensis* (**a**: Phosphorus solubilization (NIBRIP); **b**: Siderophore production (CAS); **c**: ACC deaminase enzyme activity (ACC); **d**:Nitrogen Fixation; **e**: Indole acetic acid (IAA) production).

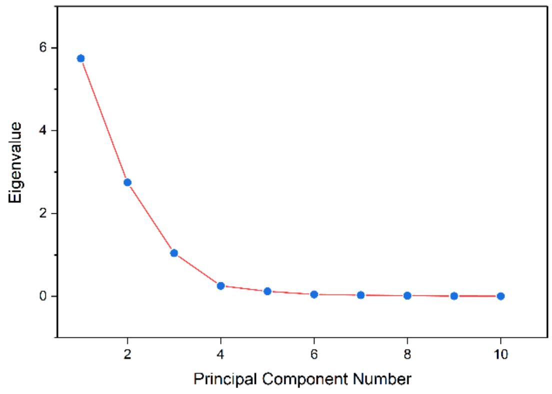

Figure S3. The PCA biplot analysis of physiological and biochemical data in *B. cereus* and *B. thuringiensis* inoculated barley plants.

Table S1. Physiological parameter variance homogeneity tests in PGPR-inoculated barley plants (SL: Shoot lenght; RL: Root lenght; BM: Biomass; DW: Dry weight).

		Levene Statistic	df1	df2	Sig.
SL	Based on Mean	1,005	11	94	,448
	Based on Median	,657	11	94	,775
	Based on Median and with adjusted df	,657	11	56,567	,772
	Based on trimmed mean	,918	11	94	,527
RL	Based on Mean	,939	11	103	,507
	Based on Median	,744	11	103	,694
	Based on Median and with adjusted df	,744	11	92,257	,694
	Based on trimmed mean	,947	11	103	,499
DW	Based on Mean	4,401	11	87	,000
	Based on Median	1,657	11	87	,097
	Based on Median and with adjusted df	1,657	11	58,263	,107
	Based on trimmed mean	4,161	11	87	,000
BM	Based on Mean	11,683	11	91	,000
	Based on Median	2,169	11	91	,023
	Based on Median and with adjusted df	2,169	11	44,806	,034
	Based on trimmed mean	10,389	11	91	,000

Table S2. The ANOVA of measured physiological parameters (*F values*) (SL: Shoot lenght; RL: Root lenght; BM: Biomass; DW: Dry weight).

		Sum of Squares	df	Mean Square	F	Sig.
SL	Between Groups	1974,922	11	179,538	56,479	,000
	Within Groups	298,814	94	3,179		
	Total	2273,736	105			
RL	Between Groups	137,311	11	12,483	8,082	,000
	Within Groups	159,089	103	1,545		
	Total	296,400	114			
DW	Between Groups	,007	11	,001	37,553	,000
	Within Groups	,002	87	,000		
	Total	,009	98			
BM	Between Groups	,866	11	,079	65,505	,000
	Within Groups	,109	91	,001		
	Total	,976	102			

Table S3. Biochemical parameter variance homogeneity tests in PGPR-inoculated barley plants (Pro: Total protein content; Chl: Total chlorophyll content; H_2O_2: Hydrogen peroxide content; TBARs: Lipid peroxidation content; POX: Peroxidase activity; CAT: Catalase activity).

		Levene Statistic	df1	df2	Sig.
Pro	Based on Mean	4,608	11	73	,000
	Based on Median	2,135	11	73	,028
	Based on Median and with adjusted df	2,135	11	43,467	,038
	Based on trimmed mean	4,577	11	73	,000
Chl	Based on Mean	1,896	11	108	,048
	Based on Median	1,412	11	108	,178
	Based on Median and with adjusted df	1,412	11	72,001	,186
	Based on trimmed mean	1,805	11	108	,062

**POX**	Based on Mean	1,926	11	51	,058
	Based on Median	,598	11	51	,822
	Based on Median and with adjusted df	,598	11	34,012	,817
	Based on trimmed mean	1,771	11	51	,084
**CAT**	Based on Mean	1,282	11	53	,260
	Based on Median	1,090	11	53	,387
	Based on Median and with adjusted df	1,090	11	23,300	,410
	Based on trimmed mean	1,210	11	53	,303
**TBARs**	Based on Mean	1,310	11	52	,246
	Based on Median	,835	11	52	,607
	Based on Median and with adjusted df	,835	11	39,749	,607
	Based on trimmed mean	1,284	11	52	,260
**H_2O_2**	Based on Mean	2,501	11	75	,010
	Based on Median	1,586	11	75	,120
	Based on Median and with adjusted df	1,586	11	23,692	,167
	Based on trimmed mean	2,571	11	75	,008

Table S4. The ANOVA of measured biochemical parameters (*F values*) (Pro: Total protein content; Chl: Total chlorophyll content; H_2O_2: Hydrogen peroxide content; TBARs: Lipid peroxidation content; POX: Peroxidase activity; CAT: Catalase activity).

		Sum of Squares	df	Mean Square	F	Sig.
**Pro**	Between Groups	15,797	11	1,436	67,221	,000
	Within Groups	1,560	73	,021		
	Total	17,357	84			
**Chl**	Between Groups	3399,114	11	309,010	56,178	,000
	Within Groups	594,059	108	5,501		
	Total	3993,173	119			
**POX**	Between Groups	81,851	11	7,441	154,553	,000
	Within Groups	2,455	51	,048		
	Total	84,306	62			

**CAT**	Between Groups	10,180	11	,925	38,269	,000
	Within Groups	1,282	53	,024		
	Total	11,462	64			
**TBARs**	Between Groups	,369	11	,034	11,818	,000
	Within Groups	,148	52	,003		
	Total	,516	63			
**H_2O_2**	Between Groups	3932,753	11	357,523	26,513	,000
	Within Groups	1011,345	75	13,485		
	Total	4944,098	86			

Table S5. The PCA percentage of variance (%) and cumulative (%).

Principal Component Number	Eigenvalue	Percentage of Variance (%)	Cumulative (%)
1	5,74172	57,41716	57,41716
2	2,7486	27,48598	84,90314
3	1,04181	10,41809	95,32123
4	0,25379	2,53795	97,85918
5	0,11863	1,1863	99,04547
6	0,04422	0,44219	99,48766
7	0,02841	0,28408	99,77174
8	0,01467	0,14674	99,91849
9	0,0054	0,05398	99,97246
10	0,00275	0,02754	100

Table S6. The correlation table of physiological and biochemical analysis data.

		SL	RL	DW	BM	Pro	Chl	POX	CAT	TBARs	H_2O_2
**SL**	Pearson Correlation	1	,200*	,790**	,756**	-,377**	,650**	-,624**	,441**	-,257	-,481**
	Sig. (2-tailed)		,043	,000	,000	,001	,000	,000	,001	,053	,000
	N	106	102	88	95	76	106	58	58	57	76
**RL**	Pearson Correlation	,200*	1	,097	,331**	-,560**	,564**	-,386**	,449**	,345**	-,278*
	Sig. (2-tailed)	,043		,354	,001	,000	,000	,002	,000	,006	,011
	N	102	115	94	99	81	115	62	60	62	84
**DW**	Pearson Correlation	,790**	,097	1	,774**	-,259*	,532**	-,651**	,404**	-,168	-,282*
	Sig. (2-tailed)	,000	,354		,000	,022	,000	,000	,001	,221	,012
	N	88	94	99	94	78	99	58	61	55	78

**BM**	Pearson Correlation	,756**	,331**	,774**	1	-,414**	,663**	-,735**	,635**	-,104	-,402**
	Sig. (2-tailed)	,000	,001	,000		,000	,000	,000	,000	,424	,000
	N	95	99	94	103	80	103	60	62	61	82
**Pro**	Pearson Correlation	-,377**	-,560**	-,259*	-,414**	1	-,716**	,600**	-,314*	-,465**	,408**
	Sig. (2-tailed)	,001	,000	,022	,000		,000	,000	,028	,000	,000
	N	76	81	78	80	85	85	51	49	54	71
**Chl**	Pearson Correlation	,650**	,564**	,532**	,663**	-,716**	1	-,592**	,657**	-,023	-,479**
	Sig. (2-tailed)	,000	,000	,000	,000	,000		,000	,000	,854	,000
	N	106	115	99	103	85	120	63	65	64	87
**POX**	Pearson Correlation	-,624**	-,386**	-,651**	-,735**	,600**	-,592**	1	-,118	-,449**	,063
	Sig. (2-tailed)	,000	,002	,000	,000	,000	,000		,482	,005	,658
	N	58	62	58	60	51	63	63	38	37	52
**CAT**	Pearson Correlation	,441**	,449**	,404**	,635**	-,314*	,657**	-,118	1	-,348*	-,581**
	Sig. (2-tailed)	,001	,000	,001	,000	,028	,000	,482		,030	,000
	N	58	60	61	62	49	65	38	65	39	52
**TBARS**	Pearson Correlation	-,257	,345**	-,168	-,104	-,465**	-,023	-,449**	-,348*	1	,130
	Sig. (2-tailed)	,053	,006	,221	,424	,000	,854	,005	,030		,340
	N	57	62	55	61	54	64	37	39	64	56
**H_2O_2**	Pearson Correlation	-,481**	-,278*	-,282*	-,402**	,408**	-,479**	,063	-,581**	,130	1
	Sig. (2-tailed)	,000	,011	,012	,000	,000	,000	,658	,000	,340	
	N	76	84	78	82	71	87	52	52	56	87

* Correlation is significant at the 0.05 level (2-tailed). ** Correlation is significant at the 0.01 level (2-tailed).

Table S7. T-test and effect values of physiological and biochemical analysis data.

T-Test

One-Sample Statistics

	N	Mean	Std. Deviation	Std. Error Mean
SL	106	31,5849	4,65345	,45198
RL	115	17,8000	1,61245	,15036
DW	99	,0682	,00959	,00096
BM	103	,6944	,09780	,00964
Pro	85	1,2885	,45457	,04930
Chl	120	41,0825	5,79276	,52880
POX	63	2,3371	1,16610	,14691
CAT	65	1,1778	,42319	,05249
TBARS	64	,5492	,09052	,01132
H2O2	87	36,7955	7,58218	,81290

One-Sample Test

	Test Value = 0					
					95% Confidence Interval of the Difference	
	t	df	Sig. (2-tailed)	Mean Difference	Lower	Upper
SL	69,881	105	<,001	31,58491	30,6887	32,4811
RL	118,381	114	<,001	17,80000	17,5021	18,0979
DW	70,686	98	<,001	,06815	,0662	,0701
BM	72,055	102	<,001	,69438	,6753	,7135
Pro	26,133	84	<,001	1,28848	1,1904	1,3865
Chl	77,689	119	<,001	41,08250	40,0354	42,1296
POX	15,908	62	<,001	2,33714	2,0435	2,6308
CAT	22,439	64	<,001	1,17785	1,0730	1,2827
TBARS	48,537	63	<,001	,54922	,5266	,5718
H2O2	45,265	86	<,001	36,79552	35,1795	38,4115

One-Sample Effect Sizes

				95% Confidence Interval	
		Standardizer[a]	Point Estimate	Lower	Upper
SL	Cohen's d	4,65345	6,787	5,850	7,722
	Hedges' correction	4,68703	6,739	5,808	7,667
RL	Cohen's d	1,61245	11,039	9,595	12,480
	Hedges' correction	1,62316	10,966	9,532	12,398
DW	Cohen's d	,00959	7,104	6,090	8,115
	Hedges' correction	,00967	7,050	6,044	8,053
BM	Cohen's d	,09780	7,100	6,107	8,090
	Hedges' correction	,09853	7,047	6,062	8,030
Pro	Cohen's d	,45457	2,835	2,355	3,310
	Hedges' correction	,45868	2,809	2,334	3,281
Chl	Cohen's d	5,79276	7,092	6,173	8,008
	Hedges' correction	5,82959	7,047	6,134	7,958
POX	Cohen's d	1,16610	2,004	1,571	2,431
	Hedges' correction	1,18044	1,980	1,552	2,402
CAT	Cohen's d	,42319	2,783	2,242	3,320
	Hedges' correction	,42823	2,750	2,215	3,281
TBARS	Cohen's d	,09052	6,067	4,980	7,150
	Hedges' correction	,09162	5,995	4,921	7,064
H2O2	Cohen's d	7,58218	4,853	4,097	5,605
	Hedges' correction	7,64912	4,810	4,062	5,556

a. The denominator used in estimating the effect sizes.
Cohen's d uses the sample standard deviation.
Hedges' correction uses the sample standard deviation, plus a correction factor.
